# The Role of Biopsy in the Workup of Patients with Neuroblastoma: Comparison of the Incidence of Surgical Complications and the Diagnostic Reliability of Diverse Techniques

**DOI:** 10.3390/children8060500

**Published:** 2021-06-12

**Authors:** Irene Paraboschi, Ester Bolognesi, Adele Giannettoni, Stefano Avanzini, Michele Torre, Giuseppe Martucciello

**Affiliations:** 1 Wellcome/EPSRC Centre for Interventional & Surgical Sciences, University College London, London WC1E 6BT, UK; 2 DiNOGMI “Dipartimento di Eccellenza”, University of Genoa, 16132 Genoa, Italy; esterbolognesi94@gmail.com (E.B.); martucciello@yahoo.com (G.M.); 3 Department of Paediatric Surgery, IRCCS Giannina Gaslini Children’s Hospital, 16147 Genoa, Italy; stefanoavanzini@gaslini.org (S.A.); MicheleTorre@gaslini.org (M.T.); 4 Dipartimento di Medicina Sperimentale e Chirurgia, University of Rome Tor Vergata, 00133 Rome, Italy; adele.giannettoni@gmail.com

**Keywords:** neuroblastoma, pediatric surgery, open incisional biopsy, minimally invasive thoracoscopic incisional biopsy, minimally invasive laparoscopic incisional biopsy, ultrasound-guided core needle biopsy, laparoscopic-assisted core needle biopsy

## Abstract

Neuroblastoma (NB) is the most common extracranial solid tumor in childhood, accounting for approximately 15% of all cancer-related deaths in the pediatric population. The overall survival of children with high-risk disease is around 40–50% despite the aggressive treatment protocols. In accordance with the most recent guidelines, a complete classification of the primary tumor, including its histopathological and molecular analysis, is necessary. In this regard, the biopsy of the primary tumor is an important diagnostic procedure adopted not only to confirm the diagnosis but also for staging and risk stratification of the disease. In this study, the authors describe their unicentric experience with four different approaches adopted for sampling NB tumors: (i) the open incisional biopsy; (ii) the minimally invasive thoracoscopic/laparoscopic incisional biopsy; (iii) the ultrasound-guided core needle biopsy; (iv) the laparoscopic-assisted core needle biopsy. The benefits of each technique are analyzed along with their contraindications.

## 1. Introduction

Neuroblastoma (NB) is the most common extracranial solid tumor in children [1]. It is characterized by a an extensive clinical pattern and adverse outcomes despite aggressive diagnostic and therapeutic workup of patients [1,2].

In accordance with the most recent guidelines, a complete classification of the primary tumor, including a histopathological [3] and molecular [4,5,6,7,8] analysis is required.

The biopsy of the primary tumor is an important diagnostic procedure adopted not only to confirm the diagnosis, but also for staging and risk stratification of the disease [3]. Based on the International Neuroblastoma Risk Group (INRG) classification system—which takes into account age at diagnosis, histopathological features, MYCN status, DNA index and segmental chromosomal aberrations (SCA)—patients can be placed into a low-, intermediate- or high-risk group. The resulting stratification determines the clinical approach, defining both the intensity and the duration of the treatment [9].

To date, several techniques have been reported to obtain samples from the primary tumor [10,11,12]. They range from invasive procedures, such as the open incisional biopsy, to minimally invasive procedures, such as those thoracoscopically/laparoscopically assisted or ultrasound-guided.

In this study, the authors describe four different approaches adopted for sampling NB tumors, together with retrospectively reviewing their unicentric experience. Particular emphasis was given to the comparison of the different techniques concerning the incidence of perioperative complications and the rate of inadequate tissue for histopathological and molecular characterization.

## 2. Material and Methods

Four different biopsy techniques for NB tissue acquisition are described in detail: (i) the open incisional biopsy; (ii) the minimally invasive thoracoscopic/laparoscopic incisional biopsy; (iii) the ultrasound-guided core needle biopsy; (iv) the laparoscopic-assisted core needle biopsy. The benefits of each technique are analyzed along with their contraindications.

Moreover, a retrospective review of 48 patients undergoing biopsy for diagnosis and risk stratification of NB at the Giannina Gaslini Children’s Hospital in Genoa, Italy, from January 2009 to December 2019 was reported.

From this study, those patients who underwent tumor biopsy in other centers were excluded, as well as patients with stage 4S disease.

A comparison of the incidence of perioperative complications and tissue inadequacy for fully characterizing the primary tumor (both histopathologically and molecularly) was performed comparing between the different biopsy techniques.

The one-way analysis of variance (ANOVA) was used to determine whether there were any statistically significant differences between the incidence of perioperative complications and tissue inadequacy for fully characterizing the primary tumor (both histopathologically and molecularly), comparing the four independent groups of patients.

## 3. Results

### 3.1. Description of the Biopsy Techniques

*(i)*  *Open Incisional Biopsy*

This represents traditional surgery involving a skin incision and a direct manipulation of the viscera by the operating surgeon, using the transperitoneal approach to reach the tumor mass. This technique is usually adopted when the tumor is too difficult to reach through less-invasive techniques or when the mass is located in easily accessible sites (such as underneath the navel or the abdominal wall). Otherwise, it is adopted when a core needle biopsy had been inadequate for diagnostic purposes.

By adopting the open incisional biopsy, a small portion of the mass is sampled together with the scissors, which are placed in a test tube containing the culture media. The hemostasis of the primary tumor is achieved by electrifying the scissors, allowing a cauterization of the excised area.

*(ii)*  *Minimally invasive thoracoscopic/laparoscopic incisional biopsy*

This technique represents an evolution of the previous one. The surgical procedure is carried out endoscopically under the guidance of a special camera, using surgical instruments with a diameter of 3–12 mm. The minimally invasive approach can be performed both in the chest (thoracoscopy) or in the abdomen (laparoscopy). Once the mass is reached through the minimally invasive tools, endoscopic scissors are used to sample the primary lesion. They can be electrified to cauterize the surgical site providing an accurate hemostasis.

The benefits of this technique rely on the possibility of sampling the primary tumor through a less-invasive approach, in this way reducing the intraoperative time and the hospital stay together with a clearer view of the surgical field obtained through the use of the scope.

In selected cases (when intraoperative complications occur or when the tumor is too difficult to be reached with minimally invasive tools), this invasive approach can be easily and quickly converted into the more traditional open approach.

The choice between these two techniques depends on the general condition of the child, his/her weight, his/her age, the presence of image-defined risk factors, the topography and the size of the primary tumor [13,14].

Adrenal NB tumors represent the typical indication to perform a laparoscopic incisional biopsy, since they are usually too deep to be sampled through an open or a percutaneous core needle biopsy.

*(iii)*  *Ultrasound-guided core needle biopsy*

This technique has become one of the most common diagnostic procedures adopted in pediatric oncology, due to its safety and efficacy.

**Figure 1:**
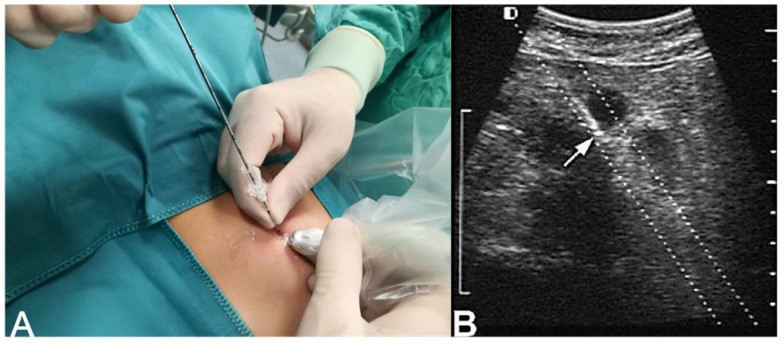
(**A**). Ultrasound scan of a large adrenal tumor. (**B**). Ultrasound-guided core needle biopsy (the arrow shows the tip of the needle within the adrenal gland).

The neoplasm is identified with an ultrasound (US) scan. Under US guidance, a mandrel is placed into the lesion, preventing the contamination of the abdominal organs with biopsied specimens. Once the target has been identified, the 16G Stericut device is inserted into the mandrel and several tumor biopsies can be obtained. Moreover, the mandrel can be angulated differently in order to sample different tumor areas (Figure 1).

The main benefit of this procedure is the quick recovery time involved.

Absolute contraindications to this procedure are the presence of critical anatomical relationships between the tumor and the abdominal organs, or patients’ uncorrectable coagulopathies [14]. Relative contraindications include major comorbidities, such as hemodynamic or respiratory instabilities [14].

*(iv)*  *Laparoscopically assisted core needle biopsy*

**Figure 2:**
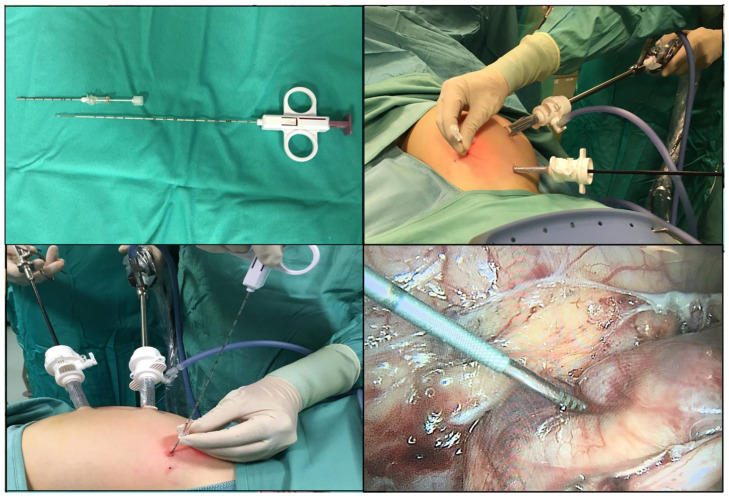
Laparoscopically assisted core needle biopsy (Group iv). Under laparoscopic vision, a mandrel is inserted through the abdominal wall into the tumor. Hence, the Stericut device is placed through the mandrel and the tumor biopsy is obtained. The procedure can be repeated several times and the position of the mandrel can be modified in order to sample multiple areas of the primary tumor. Then the tumor biopsy is collected sterile and placed in a test tube containing culture media.

This procedure combines the minimally invasive access to the primary tumor with the core needle biopsy technique (Figure 2).

Thanks to the minimally invasive nature of this procedure, it is possible to identify the mass and to guide the trajectory of the introducer spindle under endoscopic guidance.

Thanks to the full-core mechanism with coaxial introducer, it is possible to sample multiple portions of the primary tumor with different depths and angles [11].

The benefits of this surgical technique rely on obtaining a large quantity of tissue, at the same time maintaining a minimal invasiveness and a high diagnostic accuracy.

This technique is ideal to achieve a full characterization of the primary mass, especially with regards to its molecular biology, which can be heterogenous within the tumor.

The limiting factors of this technique are related to the difficulty of performing an accurate hemostasis in case of massive bleeding. However, the conversion into an open procedure is always feasible.

### 3.2. Retrospective Analysis of the Series

Forty-eight patients (28 females, 20 males) underwent a NB tumor biopsy at the pediatric surgery division of the Giannina Gaslini Children’s Hospital in the study period. The mean patient age was five years (range: 1 month–17 years).

Thirty-seven (77.1%) patients had an abdominal tumor, six (12.5%) had a thoracic tumor, two (4.2%) had a cervical tumor and three (6.3%) had a pelvic NB tumor.

In three (6.3%) patients, a single tumor sample was taken; in twenty-two (46.8%) patients, from two to four tumor samples were taken; in twenty-two (46.8%) patients, more than five tumor samples were taken referring to patients belonging to groups (iii) and (iv).

Of the 48 patients:

- 6 (12.5%) cases underwent an open incisional biopsy, group (i);
- 5 (10.4%) cases underwent a minimally invasive thoracoscopic/laparoscopic incisional biopsy, group (ii);
- 18 (37.5%) cases underwent an ultrasound-guided core needle biopsy, group (iii);
- 19 (39.6%) cases underwent a laparoscopically assisted core needle biopsy, group (iv).

### 3.3. Perioperative Complications

One (16.7%) patient in group (i) developed an intraoperative complication consisting in a duodenal perforation. No complications occurred in the group (ii). One (5.6%) patient in group (iii) developed an ipsilateral pleural effusion was which was conservatively treated. One (5.3%) patient in group (iv) developed postoperative hematuria which was conservatively treated with intravenous infusion therapy and an indwelling bladder catheter for 48 h.

### 3.4. Histopathological Analysis

The specimen material was considered inadequate for histopathological analysis in one (16.7%) patient in group (i), in zero (0.0%) patients in group (ii), in two (11.0%) patients in group (iii) and in zero (0.0%) patients in group (iv).

Overall, thirty-eight (79.2%) neuroblastomas, three (6.3%) ganglioneuromas and four (8.3%) ganglioneuroblastomas were diagnosed by tumor biopsy.

### 3.5. Molecular Characterization

The molecular characterization of the primary tumor was not performed in three cases as the histopathological analysis showed: a mature ganglioneuroma (*n* = 2) and a mature lipomatous ganglioneuroma (*n* = 1).

The specimen material was considered adequate for the molecular characterization of the primary tumor in all the other cases.

Numerical chromosome aberrations were reported in seven (14.6%) patients, segmental chromosome aberrations (SCA) were present in eighteen patients (37.5%) and MYCN amplification was detected in five (10.4%) patients.

### 3.6. Statistics

No statistically significant difference was found between the four groups of patients regarding the incidence of perioperative complications (*p*-value = 0.7046) or the incidence of inadequate specimens for histopathological (*p*-value = 0.3412) or molecular (*p*-value = 1.000) characterization.

## 4. Discussion

Neuroblastoma (NB) is characterized by heterogeneous clinical behavior in neonates and often adverse outcomes in toddlers [1]. In fact, while NB can regress spontaneously without intervention in newborns, older children can succumb after months or years of strenuous treatment [15].

The histological and molecular features of the primary tumor can vary from patient to patient, and therapeutic choices depend on appropriate tissue sampling and risk classification [16]. It is therefore paramount to appropriately risk-stratify patients to ensure an optimal treatment approach [17].

In this regard, a variety of clinical and biologic factors have been identified and traditionally used by the Children’s Oncology Group (COG): age at diagnosis, INSS (International Neuroblastoma Staging System) to define the extent of disease, INPC (International Neuroblastoma Pathology Classification) tumor histology criteria, MYCN status, DNA index and segmental chromosomal aberrations (SCA) [18].

In particular, MYCN amplification and SCA are the most important indicators that define the overall disease risk, guiding the treatment decisions [4,5,6,7,8].

Based on International Neuroblastoma Risk Group (INRG) classification system, patients can be placed on a low-, intermediate- or high-risk group, which determines both the intensity and the duration of the treatment [9]. Currently available strategies include surgery, chemotherapy, radiotherapy and immunotherapy. Lately, these methods have been coupled with strategies targeting specific oncogenic drivers of neuroblastoma, including MYCN, ALK, and TrkB, that are associated with HR-NB, and novel treatment strategies are currently being investigated [19]. It has been extensively demonstrated that surgical interventions, radiotherapy and chemotherapy result in potentially detrimental effects ranging from hearing loss, cataracts, dental disease, endocrinopathies, mental retardation, cardiac and renal toxicity [20].

While the future of NB treatment appears to be characterized by tailored therapies based on individual genetic predisposition [21,22,23], it is nowadays still fundamental to ensure an appropriate histopathological diagnosis in order to optimize the clinical outcome, reducing exposure to treatment related toxicity.

Therefore, the optimal method for tissue sampling should allow for adequate tissue for complete histopathological and molecular characterization, at the same time minimizing patient morbidity [24,25].

Mullassery et al. conducted a retrospective review of medical case records of children diagnosed with neuroblastoma at a single center, comparing the utility of open biopsy and image-guided needle biopsy in supporting the definitive diagnosis and risk stratification. Image-guided needle biopsy was found to yield adequate tissue sampling, resulting in appropriate diagnosis, risk classification and staging of neuroblastoma [26].

In a retrospective review of patients who underwent biopsy for intermediate- and high-risk neuroblastoma, Campagna et al. compared the safety, sample adequacy and therapeutic course of percutaneous core needle biopsy versus surgical biopsy. The outcomes were compared with results from a previous study conducted by Hassan et al. at the same institution, suggesting that percutaneous core needle biopsy can be considered a valid alternative to surgical biopsy when both methods are indicated. It also appears that the emerging use of percutaneous core needle biopsy and minimally invasive surgical techniques provide an accurate diagnosis and minimize complications without compromising treatment standards [26,27,28].

Overman et al. presented a multi-institutional retrospective study performed by the Pediatric Surgical Oncology Research Collaborative on children with neuroblastoma at 12 institutions over a 3-year period. Percutaneous core needle biopsy resulted as a feasible alternative to incisional biopsy for selected patients. Although the latter was found to be superior in determining tumor ploidy status and LOH at 11q, PCNB ability to determine MYCN copy number was perfectly comparable to incisional biopsy. Nevertheless, despite the validity of percutaneous core needle biopsy, the authors concluded that it remains inadequate for obtaining important molecular data in a significant fraction of patients [25].

The concept of minimizing patient morbidity should guide the choice of the operating surgeon, who should also consider the topographic anatomy of the primary lesion, its anatomical relationship with some critical structures based on the image-defined risk factors (IDRF) and his/her own surgical experience [14,27].

In the last two decades, open biopsies have decreased in number due to the longer postoperative course which is usually associated with a delay in starting the adjuvant chemotherapy.

At the same time, the ultrasound-guided procedures have been progressively abandoned due to the limits of the operator subjectivity and the difficulty in controlling intraoperative bleeding.

Conversely, the laparoscopically assisted core needle biopsy has been preferred as it allows the operating surgeon to sample different portions of the primary tumor, prerequisite for a complete characterization of structurally heterogeneous neoplasms.

In fact, the full-core mechanism with coaxial introducer is an ideal system for a full characterization of the primary tumor. Since NB can present with a heterogeneous internal microscopic architecture with hemorrhagic or necrotic areas, performing multiple biopsies through a Stericut device is particularly useful. This aspect is also very important for multinodular NBs which have areas with different histology and heterogenous genomic alterations [3]. Interestingly, in our cohort of patients, four (50.0%) out of eight multinodular neuroblastomas were diagnosed by using the video-assisted Stericut technique.

Moreover, the laparoscopically assisted core needle biopsy provides an optimal view of the surgical field to treat possible intraoperative complications.

Regarding the occurrence of perioperative complications, no significant difference was observed in our group of patients. However, the most common occurred during the open incisional biopsy (16.7%), followed by the ultrasound-guided core needle biopsy (5.6%). Interestingly, no intra- or post-operative complications occurred in the group of patients undergoing minimally invasive procedures.

Regarding the tumor biology, both the histopathological and molecular characterization of the neoplasm are fundamental for the risk stratification of the disease [29].

In our population, even if not statistically significant, the open incisional biopsies have the highest failures in terms of tissue adequacy for histopathological analysis (16.7%), followed by the ultrasound-guided core needle biopsy (11.0%). All the minimally invasive procedures allowed a histological diagnosis. This result was encouraging considering the 10% inadequacy of the biopsies reported in literature [30]. Molecular studies were possible in all the cases investigated.

On a statistical analysis, however, no superiority was found between the different procedures in terms of incidence of perioperative complications (*p*-value = 0.7046), or in the failure rate for histopathological characterization (*p*-value = 0.3412).

In conclusion, the biopsy of the primary tumor has a fundamental role for diagnostic and prognostic purposes in case of NB.

With the limit of the small number of patients involved in this unicentric study, the main value of this article is based on highlighting the fundamental role of tumor biopsy for diagnostic and prognostic purposes in case of NB.

This is an important topic, as the role of some bioptic techniques for these tumors is still controversial. In the worldwide literature, there is a need for more data and specifics on techniques for obtaining adequate tissue. A larger international prospective pediatric registry would be useful to validate formal guidelines because the present data, collected from an Italian National Referral Centre, has provided interesting results.

Increasing the prospective recruitment of patients could cast new light on the role of tumor biopsy in children affected by NB, thus determining more adequate sample techniques.

Regarding biopsy preference, the experience of the operating surgeon should be taken into consideration as well as the topographical location of the primary tumor, together with the pros and cons of each procedure.

## Data Availability

All of these data have been collected and registered on an electronic database (Microsoft Excel 2007, Redmond, WA, USA) kept in IGG secure computer. The archiving length will be 25 years. Space for archiving hard copy files has been sufficient. No external archiving unit has been required. Professor G. Martucciello is responsible for data collection, recording and quality.
